# Regular and irregular astigmatism of bullous keratopathy using Fourier harmonic analysis with anterior segment optical coherence tomography

**DOI:** 10.1038/s41598-022-22144-w

**Published:** 2022-10-25

**Authors:** Lily Wei Chen, Takashi Ono, Yumi Hashimoto, Miki Tsuneya, Yuito Abe, Takashi Omoto, Yukako Taketani, Tetsuya Toyono, Makoto Aihara, Takashi Miyai

**Affiliations:** Department of Ophthalmology, Graduate School of Medicine, The University of Tokyo, 7-3-1, Hongo, Bunkyo-ku, Tokyo, 113-8655 Japan

**Keywords:** Medical research, Eye diseases

## Abstract

Bullous keratopathy (BK) is known to present with corneal edema and Descemet's folds, which can cause corneal astigmatism. However, no report quantitatively evaluated BK astigmatism by separating it into regular and irregular astigmatism. This study investigated the regular and irregular astigmatism of the anterior and posterior corneal surface with Fourier harmonic analysis and anterior segment optical coherence tomography. Preoperative data from 43 eyes of 41 BK patients who received corneal endothelial transplantation were compared with the data from 43 eyes of 43 subjects without corneal disease. Anterior and posterior cylinder power, central corneal thickness (CCT) and thinnest corneal thickness were significantly greater in BK. With Fourier harmonic analysis, BK eyes were found to have significantly larger anterior and posterior regular astigmatism, asymmetry component and higher-order irregularity. Asymmetry component and higher-order irregularity that accounted for the posterior irregular astigmatism increased as CCT increased in BK. Higher-order irregularity in the posterior cornea also positively correlated with worsening best corrected visual acuity. Subgroup analysis found significant correlations between CCT and posterior higher-order irregularity for intraocular surgery and laser iridotomy, but not Fuchs endothelial corneal dystrophy. This study has significance in that it revealed the characteristics of the corneal posterior irregular astigmatism of BK.

## Introduction

Bullous keratopathy (BK) is a condition that results from damages to the corneal endothelium, a monolayer of cells located on the posterior surface of the cornea. The corneal endothelial cells have focal tight junctions and Na^+^/K^+^-ATPases that help to maintain the cornea in a dehydrated state^1,2^. With decreased corneal endothelial cell density, there may be stromal edema and bullae formation in the subepithelial and epithelial layers. Causes of BK include cataract surgery, trabeculectomy, laser iridotomy, Fuchs’ endothelial corneal dystrophy, penetrating or blunt trauma, and viral infections^3–5^. Patients can develop severe vision loss and possibly pain with disease progression. The gold standard treatment is a corneal transplantation although several options are available for symptom management^6–10^.

The BK cornea under the slit-lamp may reveal corneal edema and Descemet’s folds^11^, which are pathological changes that can increase the irregularity of the anterior and posterior surfaces. There are also BK patients with poor vision despite corneal transparency, suggesting that another factor such as a distortion in corneal shape could have caused the decreased visual acuity. Currently, several methods are used in clinical practice for the evaluation of BK. These include the most basic evaluation with slit-lamp examination for signs such as corneal subepithelial bullae and stromal swelling, specular microscopy to check for decreased endothelial cell density, and ultrasonic pachymetry to determine the corneal thickness, which is also effective in advanced cases as an indicator of corneal endothelial function^12^. However, these findings represent gross changes that cannot be used to determine details regarding the corneal shape or corneal refraction. The slit lamp examination may be prone to operator dependent variability, and the confirmation of decreased endothelial cell density with a specular microscope may be effective in the early stages, but imaging becomes difficult with disease progression. While the refractive power can be evaluated using keratometry and corneal topography with Scheimpflug imaging, these cannot evaluate the back of the cornea in advanced cases^13^. Aside from the shortage of information regarding the posterior cornea, previous studies have focused on the postoperative topographic changes associated with corneal transplantation instead of BK itself^14–18^. In addition, no report quantitatively assessed the astigmatism of BK by separating it into regular and irregular astigmatism.

Fourier harmonic analysis of topographic data is a method that quantitatively separates regular and irregular astigmatism. It deconstructs raw corneal power data into a combination of periodic trigonometric functions and reconstructs these into four components: spherical component, regular astigmatism, asymmetry component, and higher-order irregularity^19–21^. This method has been used for investigating the optical quality of the cornea with cataract surgery^22–24^, trabeculectomy^25^, scleral buckling^26^, vitrectomy^27^, penetrating keratoplasty^28,29^, photorefractive keratectomy^30–32^, laser in situ keratomileusis^33^, orthokeratology^34^, keratoconus^35–37^, and pterygium^38^.

The early studies on Fourier harmonic analysis used topographic data obtained from videokeratography. However, advancements in OCT technology later made it possible to use anterior segment optical coherence tomography (AS-OCT) to obtain topographic data for both the anterior surface and the posterior surface of the cornea. Fourier harmonic analysis with AS-OCT has been used in recent years to evaluate the topographic changes associated with primary and recurrent pterygium^39^, ptosis surgery^40^, penetrating keratoplasty for keratoconus^41^, rigid gas permeable contact lens use for keratoconus^42^, and Fuchs endothelial corneal dystrophy (FECD)^43^. A study also analyzed the relationship between regular astigmatism pattern and irregular astigmatism in normal subjects^44^. AS-OCT utilizes long-wavelength infrared light that can be used to assess the posterior surface of the cornea even in eyes with corneal opacity^45^. This is an advantage over Scheimpflug corneal tomography, which uses visible light that cannot pass through the opaque cornea. The components of Fourier harmonic analysis can be useful references for decisions regarding the surgical timing and the surgery method for BK if a relationship is found with increases in corneal thickness or decreases in visual acuity. Since spectacles can correct the spherical component and regular astigmatism but not the asymmetry component and higher-order irregularity, an understanding of the regular and irregular astigmatism associated with BK through Fourier harmonic analysis can help with clinical decision making.

In this study, we evaluated the anterior and posterior corneal astigmatism of BK by Fourier harmonic analysis of corneal topography with AS-OCT. We also looked at the relationship between central corneal thickness (CCT), best corrected visual acuity (BCVA) and each component of the Fourier harmonic analysis.

## Results

### Patient demographics

Forty-three eyes of 43 subjects without corneal disease as control and 43 eyes of 41 patients with BK were analyzed in this study. The average age was 69.3 ± 9.3 years and 73.3 ± 11.8 years, respectively. There was no statistically significant difference between the 2 groups for age, gender, and right eye to left eye ratio. The BCVA was − 0.06 ± 0.04 for the control group and 1.15 ± 0.49 for the BK group (p < 0.0001). Of the 43 BK eyes, 17 (39.5%) were the result of trabeculectomy, 4 (9.3%) were the result of cataract surgery, 17 (39.5%) were caused by laser iridotomy, and 5 (11.6%) were due to FECD (Table 1).

**Table 1:** Demographic characteristics, BCVA and AS-OCT measurements of control and BK groups.

	Control group	BK group	p-value
N (eyes)	43	43	–
Age (years)	69.3 ± 9.3	73.3 ± 11.8	0.99
Sex (Male:Female)	13:30	11:32	0.80
Side (Right:Left)	25:18	19:24	0.20
Best-corrected visual acuity (logMAR)	− 0.06 ± 0.04	1.15 ± 0.49	< 0.0001
Anterior averaged keratometry (D)	49.4 ± 1.7	48.5 ± 2.7	0.093
Anterior cylinder power (D)	0.9 ± 0.7	2.3 ± 1.8	< 0.0001
Posterior averaged keratometry (D)	− 6.3 ± 0.2	− 6.4 ± 0.6	0.37
Posterior cylinder power (D)	0.3 ± 0.1	0.5 ± 0.3	0.0003
Central corneal thickness (μm)	529.4 ± 14.2	747.6 ± 102.1	< 0.0001
Thinnest corneal thickness (μm)	521.0 ± 14.0	691.4 ± 87.3	< 0.0001
**Causative disease**			
Intraocular surgery	–	21 eyes (48.8%)	
Post cataract surgery	–	− 17 eyes (39.5%)	
Post trabeculectomy		− 4 eyes (9.3%)	
Post laser iridotomy	–	17 eyes (39.5%)	
Fuchs endothelial corneal dystrophy	–	5 eyes (11.6%)	

### AS-OCT measurements and Fourier components

**Figure 1:**
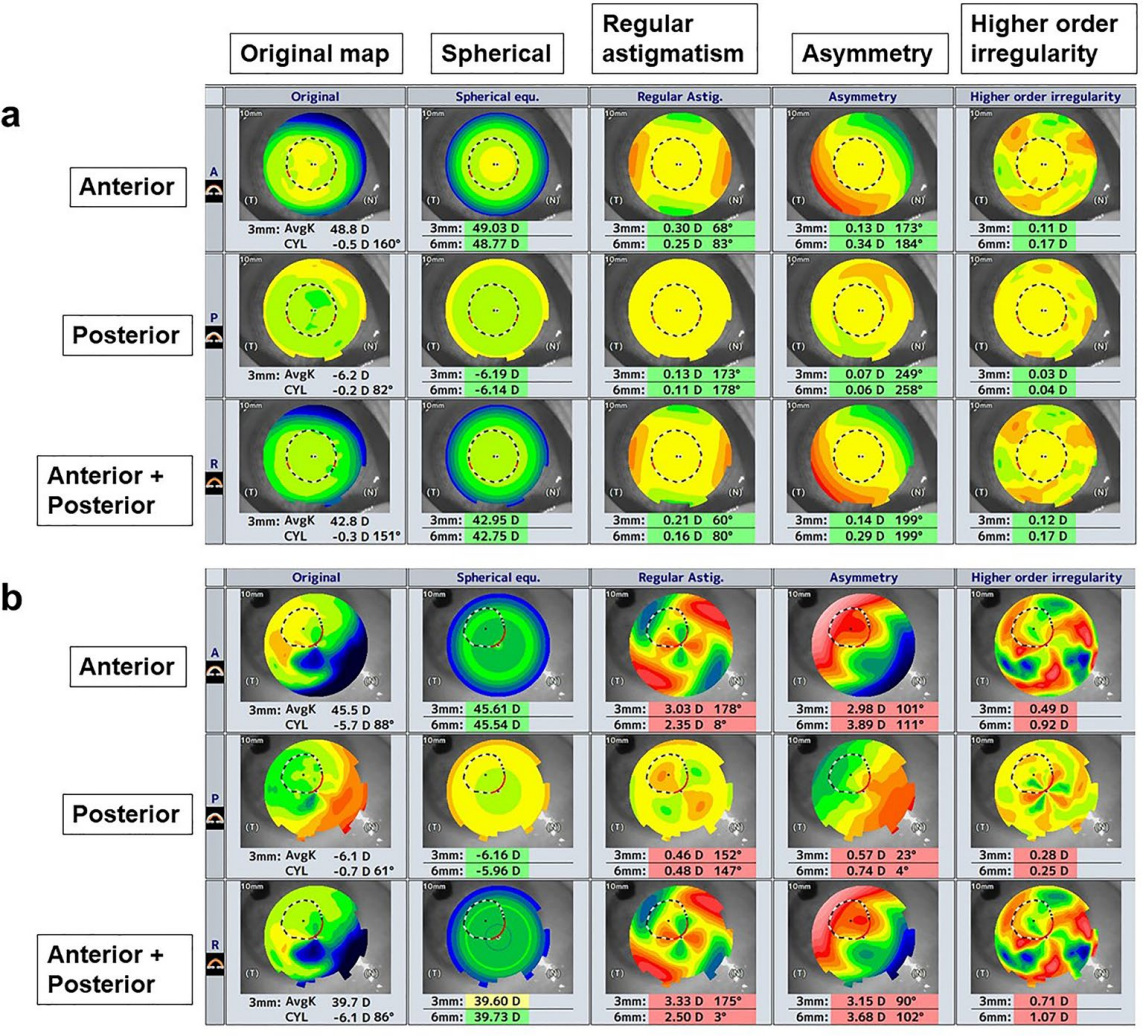
Representative Fourier maps taken with anterior segment optical coherence tomography in (**a**) control without corneal disease and (**b**) subject with bullous keratopathy (BK). The original color-coded map is separated into spherical component, regular astigmatism, asymmetry component and higher-order irregularity. Fourier indices from the central 3 mm and 6 mm zones are shown for the anterior, posterior, and anterior + posterior cornea. Central corneal thickness is 530 µm for the control and 724 µm for BK.

Anterior cylinder power, posterior cylinder power, CCT and thinnest corneal thickness were significantly greater in the BK group but there was no significant difference in the anterior or posterior averaged keratometry (p < 0.0001, p = 0.0003, p < 0.0001, p < 0.0001, respectively) (Table 1). The mean CCT was 529.4 ± 14.2 µm in the control group and 747.6 ± 102.1 µm in the BK group (p < 0.0001). Representative OCT images of the control group and the BK group are provided for comparison (Fig. 1). With Fourier harmonic analysis, the BK group was found to have significantly larger anterior and posterior regular astigmatism, asymmetry component and higher-order irregularity (p < 0.0001 for all) (Table 2). These increases were seen within the central 3 mm and 6 mm zones.

**Table 2:** Components of the Fourier harmonic analysis for the central 3 mm and 6 mm zones in control and BK groups.

Fourier components	Control group	BK group	p-value
Anterior 3 mm spherical component (D)	49.6 ± 1.8	48.7 ± 3.4	0.14
Anterior 3 mm regular astigmatism (D)	0.5 ± 0.3	1.4 ± 1.4	< 0.0001
Anterior 3 mm asymmetry component (D)	0.2 ± 0.1	1.5 ± 1.2	< 0.0001
Anterior 3 mm higher-order irregularity (D)	0.1 ± 0.1	0.6 ± 0.5	< 0.0001
Posterior 3 mm spherical component (D)	−6.3 ± 0.2	−6.2 ± 0.6	0.81
Posterior 3 mm regular astigmatism (D)	0.2 ± 0.1	0.3 ± 0.2	< 0.0001
Posterior 3 mm asymmetry component (D)	0.0 ± 0.0	0.5 ± 0.4	< 0.0001
Posterior 3 mm higher-order irregularity (D)	0.0 ± 0.0	0.3 ± 0.2	< 0.0001
Anterior 6 mm spherical component (D)	49.3 ± 1.7	48.4 ± 2.9	0.068
Anterior 6 mm regular astigmatism (D)	0.4 ± 0.3	1.3 ± 1.0	< 0.0001
Anterior 6 mm asymmetry component (D)	0.4 ± 0.2	1.7 ± 1.2	< 0.0001
Anterior 6 mm higher-order irregularity (D)	0.2 ± 0.1	0.7 ± 0.6	< 0.0001
Posterior 6 mm spherical component (D)	−6.2 ± 0.2	−6.2 ± 0.5	0.58
Posterior 6 mm regular astigmatism (D)	0.2 ± 0.1	0.3 ± 0.1	< 0.0001
Posterior 6 mm asymmetry component (D)	0.1 ± 0.0	0.6 ± 0.3	< 0.0001
Posterior 6 mm higher-order irregularity (D)	0.0 ± 0.0	0.3 ± 0.2	< 0.0001

### CCT and linear regression analysis

**Figure 2:**
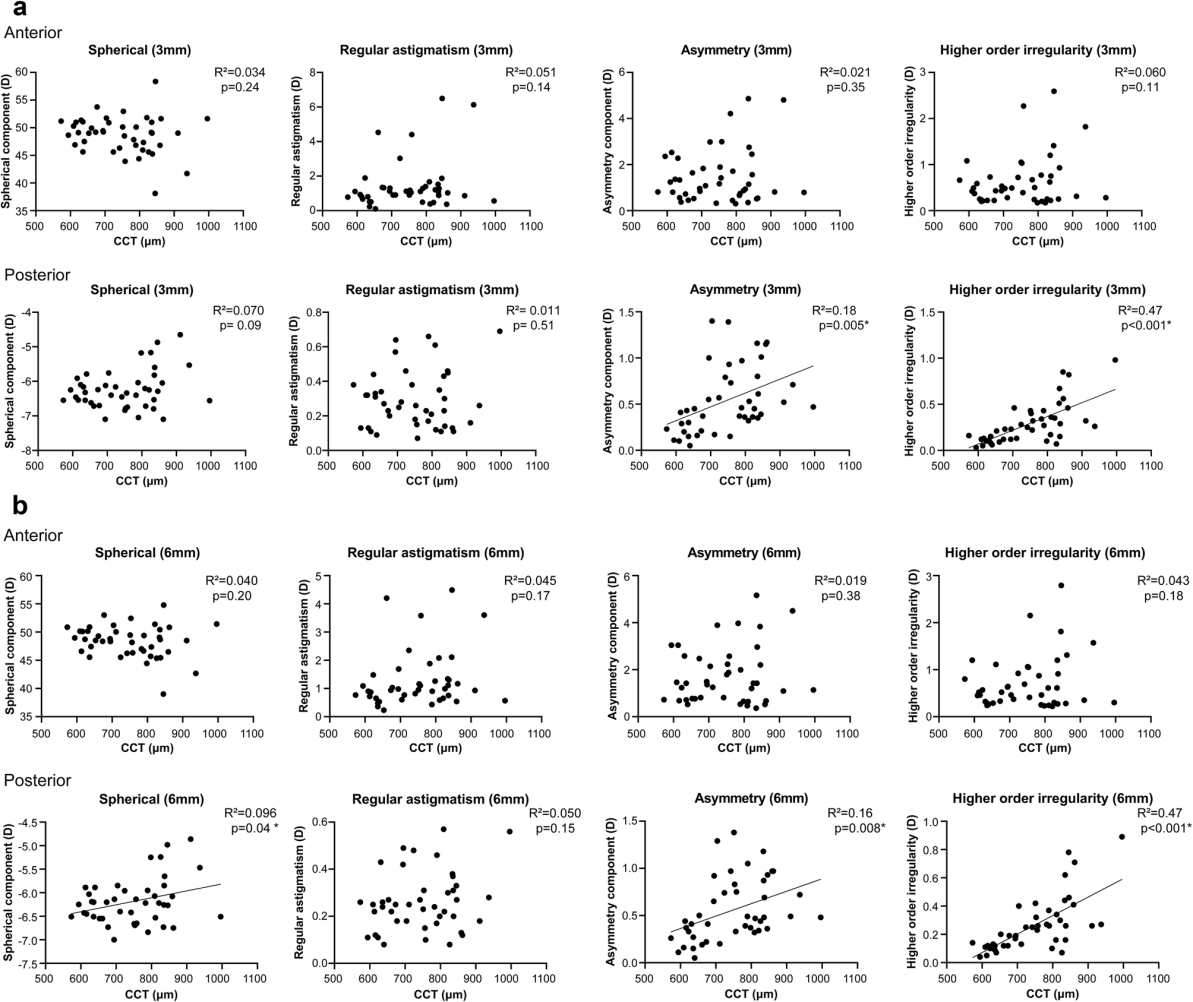
Linear regression between central corneal thickness (CCT) and each Fourier component in the anterior and posterior cornea within the central (**a**) 3 mm and (**b**) 6 mm zones. Significant correlations are seen for the posterior cornea between CCT and the spherical component within the central 6 mm zone (R^2^ = 0.096, p = 0.04), asymmetry within the central 3 mm (R^2^ = 0.18, p = 0.005), higher-order irregularity within the central 3 mm (R^2^ = 0.47, p < 0.001), asymmetry within the central 6 mm zone (R^2^ = 0.16, p = 0.008) and higher-order irregularity within the central 6 mm zone (R^2^ = 0.47, p < 0.001). Regression lines are drawn for graphs with statistical significance (*p < 0.05).

When linear regression analysis was used to check for the relationship between CCT and the 4 components of Fourier harmonic analysis in the BK group, no significant correlation was detected in the anterior cornea. With the posterior cornea, there were significant positive correlations between CCT and the spherical component within the central 6 mm zone (R^2^ = 0.096, p = 0.04), as well as asymmetry and higher-order of irregularity within the central 3 mm (R^2^ = 0.18, p = 0.005 for asymmetry; R^2^ = 0.47, p < 0.001 for higher-order irregularity) and 6 mm zones (R^2^ = 0.16, p = 0.008 for asymmetry; R^2^ = 0.47, p < 0.001 for higher-order irregularity) (Fig. 2).

**Figure 3:**
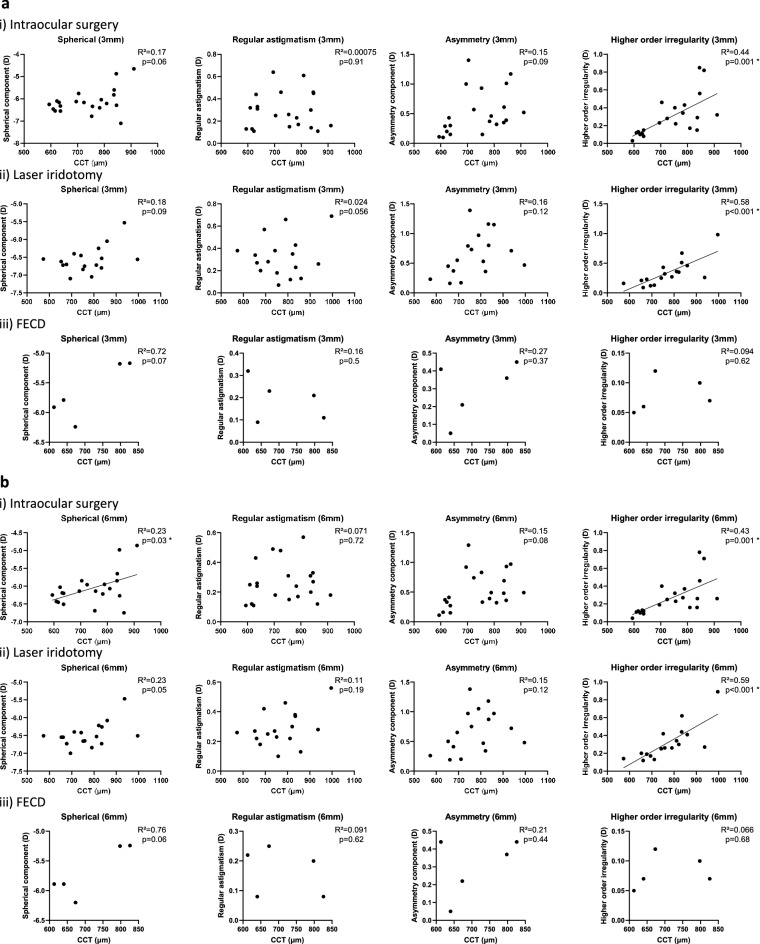
Subgroup analysis of the posterior cornea based on the cause of bullous keratopathy: intraocular surgery, laser iridotomy and Fuchs endothelial corneal dystrophy (FECD). Linear regression analysis is done between central corneal thickness (CCT) and each Fourier component within the central (**a**) 3 mm and (**b**) 6 mm zones. Linear regression shows significant correlations between CCT and the spherical component within the central 6 mm zone (R^2^ = 0.23, p = 0.03), the higher-order irregularity within the central 3 mm zone (R^2^ = 0.44, p = 0.001) and the higher-order irregularity within the central 6 mm zone (R^2^ = 0.43, p = 0.001) for the intraocular surgery group. The laser iridotomy group has significant correlations between CCT and the higher-order irregularity within the central 3 mm zone (R^2^ = 0.58, p < 0.001) and the central 6 mm zone (R^2^ = 0.59, p < 0.001). Regression lines are drawn for graphs with statistical significance (*p < 0.05).

Sub-group analysis was done after dividing the BK etiologies into 3 groups: (i) intraocular surgery—trabeculectomy or cataract surgery (21 eyes; 48.8%), (ii) laser iridotomy (17 eyes; 39.5%) and (iii) FECD (5 eyes; 11.6%). No statistical significance was seen with the anterior cornea in all the groups. For the posterior cornea, the intraocular surgery group had significant positive correlations between CCT and the spherical component within the central 6 mm zone (R^2^ = 0.23, p = 0.03), as well as the higher-order irregularity within the central 3 mm (R^2^ = 0.44, p = 0.001) and 6 mm zones (R^2^ = 0.43, p = 0.001). The laser iridotomy group had significant positive correlations between CCT and the higher-order irregularity within the central 3 mm (R^2^ = 0.58, p < 0.001) and 6 mm zones (R^2^ = 0.59, p < 0.001). No significant correlations were seen in the FECD group (Fig. 3).

### BCVA and linear regression analysis

**Figure 4:**
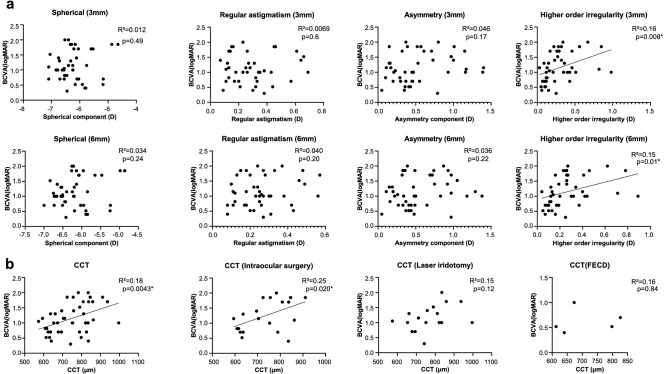
Linear regression between (**a**) each Fourier component of the posterior cornea and logMAR best corrected visual acuity (BCVA), as well as (**b**) CCT and logMAR BCVA. Significant associations are seen with higher-order irregularity in the central 3 mm zone (R^2^ = 0.16, p = 0.008) and the central 6 mm zone (R^2^ = 0.15, p = 0.01). A significant association is seen between CCT and logMAR BCVA (R^2^ = 0.18, p = 0.0043) With subgroup analysis that divides BK etiologies into three groups (intraocular surgery, laser iridotomy and FECD), only CCT in the intraoperative surgery group significantly correlates with BCVA (R^2^ = 0.25, p = 0.020). Regression lines are drawn for graphs with statistical significance (*p < 0.05).

Associations with BCVA (logMAR) were also looked at for the 4 components of Fourier harmonic analysis and CTT using linear regression analysis. No significant association was seen with the Fourier harmonic analysis in the anterior cornea. Statistically significant associations were seen with higher-order irregularity in the central 3 mm (R^2^ = 0.16, p = 0.008) and 6 mm zones (R^2^ = 0.15, p = 0.01) of the posterior cornea, as well as CCT (R^2^ = 0.18, p = 0.0043) (Fig. 4). With subgroup analysis that divided BK etiologies into three groups (intraocular surgery, laser iridotomy and FECD), only CCT significantly correlated with BCVA in the intraoperative surgery group (R^2^ = 0.25, p = 0.020).

## Discussion

This study is the first to examine the regular and irregular astigmatism in BK with Fourier harmonic analysis and AS-OCT. Fourier harmonic analysis enabled the quantitative assessment of the astigmatism, while AS-OCT provided a better assessment of the posterior cornea compared to older imaging technologies. Compared to the control group, BK eyes had increased regular and irregular astigmatism for the anterior and posterior cornea. There was no significant difference in the spherical component of the Fourier harmonic analysis between the control group and BK eyes, indicating that BK did not result in prominent protrusion or flattening. The asymmetry component and higher-order irregularity that accounted for the posterior irregular astigmatism within the central 3 mm and 6 mm zones increased as the corneal thickness increased in BK. This positive correlation was not seen in the anterior cornea. The positive correlation seen in the posterior cornea suggests that as the cornea thickens with worsening BK, the back of the cornea becomes increasingly irregular. This may be associated with Descemet folds seen on the clinical exam. Furthermore, the significant correlation between higher-order irregularity and BCVA as well as the significant correlation between CCT and BCVA point to a relationship between posterior irregular astigmatism and worsening visual function.

Other studies that looked at astigmatism in BK differed from our study in several ways regarding the study design. Yamaguchi et al. looked at the anterior and posterior corneal irregularity in 13 BK eyes that underwent Descemet-stripping endothelial keratoplasty (DSEK) using a rotating Scheimpflug camera and Zernike polynomial decomposition for corneal wavefront aberrations^14^. Although Yamaguchi et al. also found a significant increase between the control group and the BK group for preoperative anterior and posterior irregularity, the irregularity was not broken down into different components. Spadea et al. looked at 20 BK eyes that underwent DSEK with videokeratography maps and Zernike polynomial decomposition for corneal wavefront aberrations but followed the patients for a longer postoperative period than Yamaguchi et al. and investigated only the anterior irregularity^18^. Spadea et al. also found increased anterior astigmatism in the preoperative BK eyes compared to the control but did not analyze the different components that made up the astigmatism. Both studies had fewer cases than our study and did not analyze the relationship with CCT. The third study, by Oie et al., investigated the anterior and posterior cornea for FECD with Fourier harmonic analysis and AS-OCT^43^. They divided 75 eyes of 43 FECD subjects into 5 categories of severity and analyzed the association between the modified Krachmer grade or CCT and each component to see if there might be differences among groups. They also assessed the relationship between each component and BCVA using the Spearman rank correlation and found significant associations with the spherical power and asymmetry in the anterior cornea, as well as the spherical power, asymmetry, and higher-order irregularity in the posterior cornea. Their results differed from ours likely because we used a linear regression analysis, included other etiologies of BK, and had fewer FECD eyes.

The relationship between CCT and each component of the Fourier harmonic analysis was not investigated by Oie et al., so our study fills a gap within the existing literature by demonstrating significant associations between CCT and several components of the Fourier harmonic analysis in the posterior cornea.

There are two notable limitations with this study. One is that the small sample size could have decreased the statistical power and made it more difficult to detect significant findings. The effect would have been especially large with the FECD group in the subgroup analysis. The other is that this was a retrospective cross-sectional study, so how the state of the cornea changed for each subject and the exact duration of bullous keratopathy were not studied. In addition, the decision to undergo or not undergo surgery could have been influenced by the subjects’ social circumstances. In the future, it will be important to conduct a larger cohort study to acquire data at multiple time points, preferably starting before the onset of bullous keratopathy.

In conclusion, this study demonstrated that both regular and irregular corneal astigmatism increased in BK eyes compared to eyes without corneal disease. A significant correlation was seen between CCT and the spherical component, asymmetry and higher-order irregularity in the posterior cornea, and between BCVA and the higher-order irregularity of the posterior cornea. Components of the Fourier harmonic analysis did not show a correlation with CCT or BCVA in the anterior cornea. Based on these findings, advanced bullous keratopathy increases irregular astigmatism of the posterior cornea. Since posterior irregular astigmatism cannot be fully corrected with glasses or contact lenses, corneal transplantation is recommended in such cases to improve visual acuity.

## Methods

This retrospective observational study was approved by the Institutional Review Board of the Research Ethics Committee of the University of Tokyo School of Medicine (Examination No. 2020006NI) and adhered to the tenets of the Declaration of Helsinki. The need for written patient consent was waived due to the study’s retrospective nature. Information from medical charts of patients with BK who received corneal endothelial transplantation (Descemet stripping automated endothelial keratoplasty [DSAEK] or Descemet membrane endothelial keratoplasty [DMEK]) between May 2017 and August 2021 was reviewed and compared with that of age- and gender-matched subjects without corneal disease. BK was diagnosed by corneal specialists based on the clinical findings of epithelial and stromal edema, as well as a corneal endothelial density below 500 cells/mm^2^. Preoperative corneal topography was done using AS-OCT (CASIA2; Tomey Corporation, Tokyo, Japan). Patients who were not evaluated with AS-OCT during the preoperative period were excluded from the study.

BCVA was measured with decimal visual acuity and expressed in logarithm of the minimum angle of resolution (logMAR) units. CCT, Kmax, AvgK, and corneal topographic data with Fourier harmonic analysis were obtained via AS-OCT and its built-in software (CASIA2; Tomey Corporation, Tokyo, Japan). CASIA2 is capable of 10 µm axial and 30 µm transverse high-resolution imaging and has a scan depth of 13 mm, scan speed of 50,000 A-scans per second and 1310 nm wavelength light source. The corneal topography of CASIA2 analyzes the shape of the anterior and posterior corneal surfaces from high-resolution AS-OCT images. Fourier series harmonic analysis divided corneal topographic data into four components: spherical, regular astigmatism, asymmetry, and higher-order irregularity. Calculations for Fourier series harmonic analysis were as described in a previous report^19^. Dioptric powers on a mire ring i, Fi(σ ), were converted into trigonometric components as follows: Fi(σ ) = a_0_ + c_1_ cos(σ − α_1_) + c_2_ cos 2(σ − α_2_) + c_3_ cos 3(σ − α_3_) + ⋯ + c_n_ cos n(σ − α_n_ ). Spherical component is a_0_, regular astigmatism is 2c_2_, asymmetry is 2c_1_, and higher-order irregularity is the sum of c_3_…c_n_. Values of the rings that made up the central 3 mm and 6 mm zones were averaged for each parameter.

Statistical analysis was conducted with Microsoft Excel (Office 365) and Graphpad Prism 9.3.1 (Graphpad Software. San Diego, CA, USA). The unpaired t-test was used for continuous variables. The Chi-square test was used for categorical variables. p value less than 0.05 was considered statistically significant. Normal eyes from age- and gender-matched subjects were selected using the MatchIt package for R (version 3.6.1; R Foundation for Statistical Computing, Vienna, Austria). Simple linear regression analysis was done using Graphpad Prism 9.3.1 to calculate the goodness of fit (R^2^) and the statistical significance of a line with non-zero slope.

### Ethical approval

This retrospective observational study was approved by the Research Ethics Committee of the University of Tokyo School of Medicine (Examination No. 2020006NI). This study was conducted under the principles of the Declaration of Helsinki.

### Consent to participate

Due to the observational nature of the study and the use of medical records, the Institutional Review Board waived the requirement of written consent. Patients were given the opportunity to opt out of the study.

## Data Availability

The datasets analyzed during the current study are available from the corresponding author upon reasonable request.
